# Supplementary material for: Egr1 is a sex-dependent regulator of neuronal chromatin, structural plasticity, and behaviour
Source: Nat Commun. 2025 Dec 13;16:11405. doi: 10.1038/s41467-025-66217-6 (PMC12738549; doi:10.1038/s41467-025-66217-6)
Supplement: Supplementary file 1 — Supplementary Information [file 41467_2025_66217_MOESM1_ESM.pdf]

Supplementary Information

Egr1 is a sex-dependent regulator of neuronal chromatin, structural plasticity, and behaviour

Devin Rocks[1], Luisa Demarchi[1], Laila Ouldibbat[1], Eric Purisic[1], Heining Cham[2], Eduardo F. Gallo[1], John M. Greally[3], Masako Suzuki[3,4], Marija Kundakovic[1]*

*Correspondence to: mkundakovic@fordham.edu (M.K.)

This PDF file includes:

Supplementary Figures

Supplementary Figure 1. *Ppp1r1b* expression in the ventral hippocampus across the oestrus cycle.

Supplementary Figure 2. Verification of targeted Egr1 overexpression in the ventral hippocampus.

Supplementary Figure 3. The effect of prepubertal ovariectomy on anxiety-related behaviour in young adult mice.

Supplementary Figure 4. Total locomotor activity is not affected by Egr1 overexpression.

Supplementary Figure 5. Egr1 overexpression and resulting sex-dependent effects on gene expression in ventral hippocampal neurons.

Supplementary Figure 6. Egr1 gained-open chromatin in ventral hippocampal neurons.

Supplementary Figure 7. Egr1 has overlapping effects on chromatin accessibility and gene expression in ventral hippocampal neurons.

Supplementary Figure 8. Egr1 induces overlapping chromatin organizational changes in ventral hippocampal neurons with proestrus females.

Supplementary Figure 9. Overlapping elements of neuronal gene regulation by Egr1 after overexpression and across the oestrous cycle.

Supplementary Figure 10. Regions targeted by Egr1 for chromatin regulation are enriched for Egr1 binding activity.

Supplementary Figure 11. Egr1 enhancers are preferentially enriched in Egr1 vs. eGFP group comparison in ventral hippocampal neurons.

Supplementary Figure 12. No effect of sham surgery on ventral hippocampal *Egr1* expression and open field behavior**.**

Supplementary Figure 13. Isolation of neuronal nuclei using fluorescence-activated nuclei sorting (FANS).

Supplementary Figure 14. Quality control for nucRNA and sequencing libraries.

Supplementary Figure 1

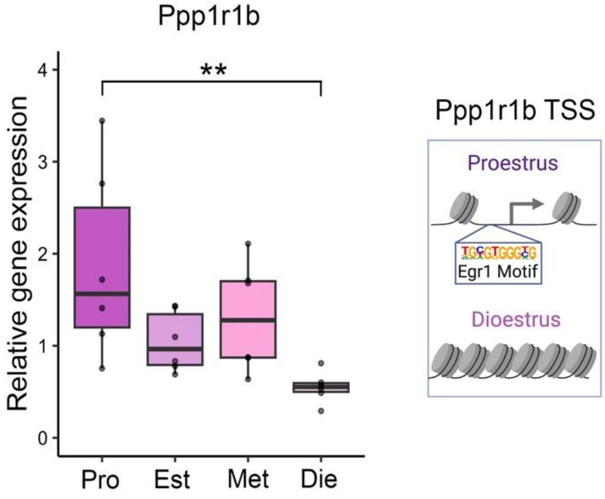

***Ppp1r1b* expression in the ventral hippocampus across the oestrus cycle.** The expression of Egr1's putative target gene *Ppp1r1b* changes in the ventral hippocampus over the oestrous cycle (left, n=5 Est, 6 Pro/Met/Die, normalized against the endogenous reference gene *Ppia*); the chromatin around the *Ppp1r1b* transcription start site (TSS), which contains the Egr1 binding motif, was previously found to be accessible in ventral hippocampal neurons of proestrus, but not dioestrus, female mice[18] (right). Box plot (box, 1st-3rd quartile; horizontal line, median; whiskers, 1.5xIQR); one-way ANOVA with Holm's post hoc test; **, P<0.01 (exact p-values provided in **Suppl. Data 1**). Pro, proestrus (purple); Est, oestrus (light-purple); Met, metoestrus (pink); Die, dioestrus (light-pink). Source data are provided as a Source Data file**.** Schematic (right) was created in BioRender. Rocks, D. (2025) https://BioRender.com/oo9h37f.

Supplementary Figure 2

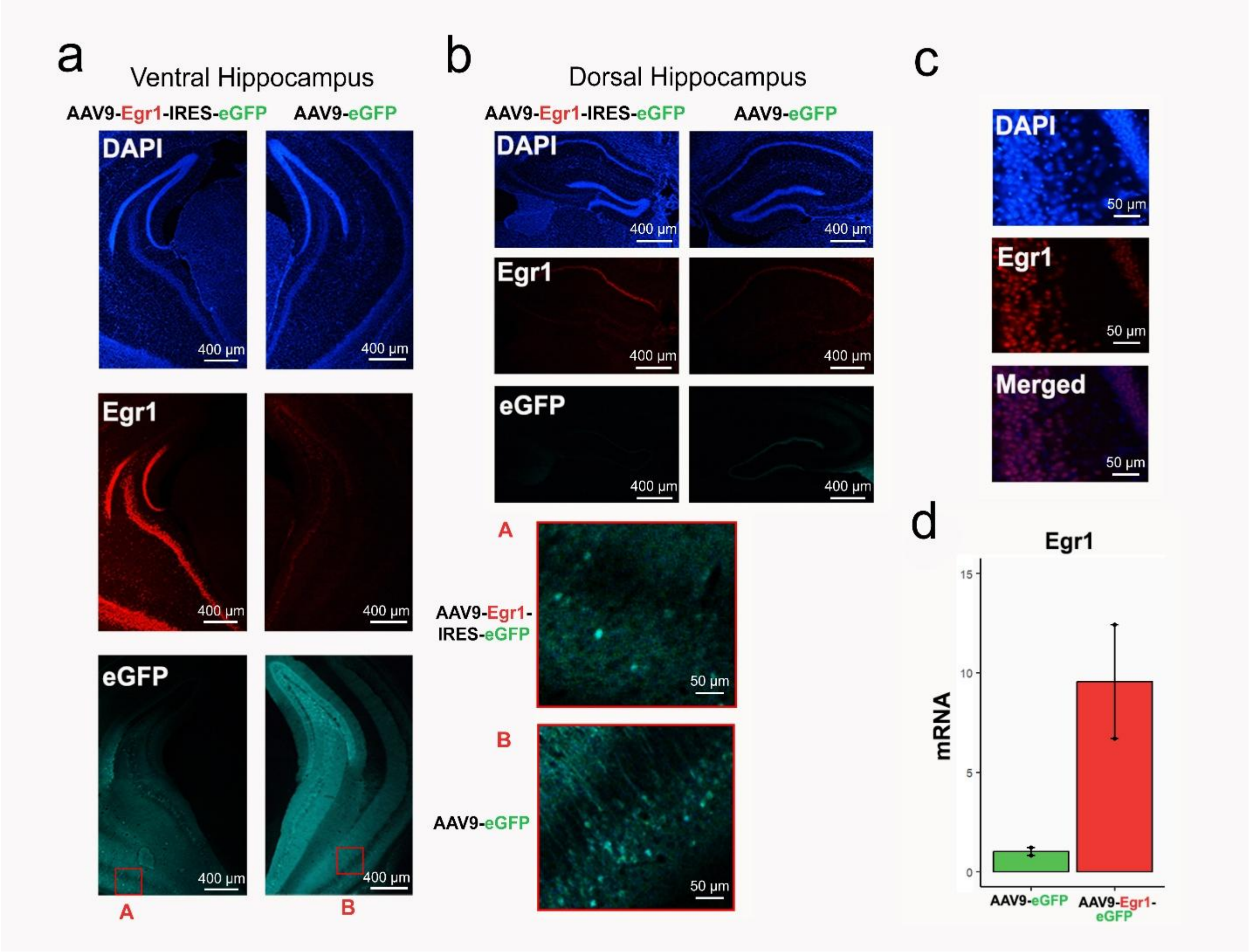

Verification of targeted Egr1 overexpression in the ventral hippocampus. a-b. Representative coronal sections containing the ventral (**a**) and dorsal (**b**) hippocampus were immunostained for Egr1 in animals that received either experimental (AAV9-Egr1-IRES-eGFP, left) or control (AAV9-eGFP, right) virus. Panels show DAPI (top), Egr1 (middle) and eGFP (bottom), with insets A and B (in red) demonstrating cellular eGFP expression in both sections. As targeted, we confirmed that Egr1 overexpression was observed in the ventral hippocampus (**a**) only, but not in the functionally distinct dorsal hippocampus (**b**). **c.** DAPI (top) and Egr1 (middle) signals overlap (bottom) in the ventral hippocampus of mice that received the Egr1 virus, confirming the nuclear localization of Egr1. Proper targeting of viral injections was verified for each animal, through either histology (behaviour cohort) or genomics data analysis (nucRNA-seq and ATAC-seq cohorts; see *Methods*) **d.** A pilot qRT-PCR experiment demonstrated *Egr1* mRNA overexpression (close to 10-fold) in the ventral hippocampus of animals injected with Egr1 virus, compared to animals that received the control virus (n=2/group), which was later confirmed with neuronal-specific nucRNA-seq (**Suppl. Fig. 5a**). Bar plots; whiskers denote standard error. Scale bars in panels a and b correspond to 400 μm, while scale bars in panel c and the insets (part of panel a) correspond to 50 μm. Source data are provided as a Source Data file**.**

Supplementary Figure 3

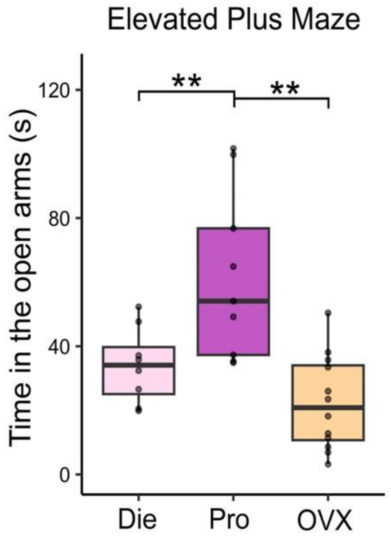

The effect of prepubertal ovariectomy on anxiety-related behaviour in young adult mice. Ovariectomized (OVX) mice that underwent ovariectomy surgery at 4 weeks of age behave similarly to low-oestrogenic, dioestrus (Die) females and show higher anxiety indices than high-oestrogenic, proestrus (Pro) females at 10 weeks of age, considering time spent in the open arms of the elevated plus maze (n=8 Die, 9 Pro, 12 OVX). Box plot (box, 1st-3rd quartile; horizontal line, median; whiskers, 1.5xIQR); one-way ANOVA with Holm's post hoc test; **, P<0.01 (exact p-values provided in **Suppl. Data 1**). Die, dioestrus (pink); Pro, proestrus (purple); OVX, ovariectomized (yellow). Source data are provided as a Source Data file**.**

Supplementary Figure 4

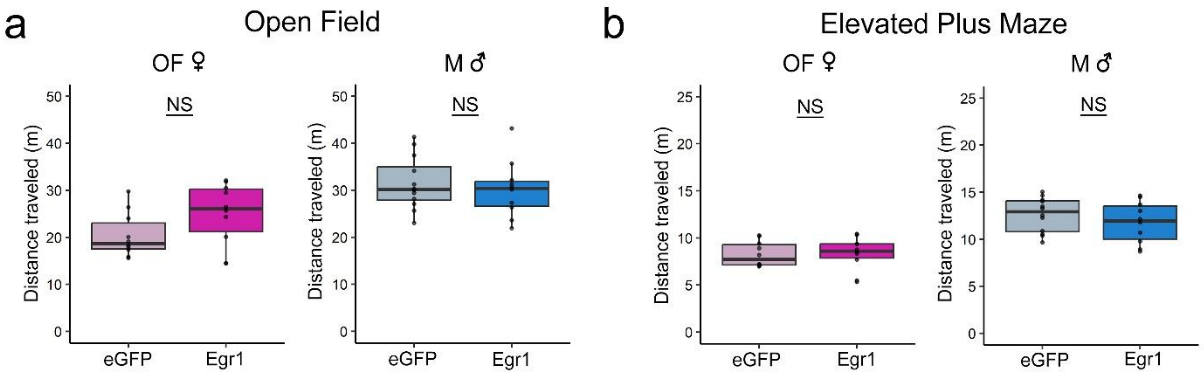

Total locomotor activity is not affected by Egr1 overexpression. Egr1 overexpression has no effect on distance travelled in either OVX females (OF, left, n=10/group) or males (M, right, n=10 Egr1, 12 eGFP) in the open field (**a**) or elevated plus maze (**b**) tests. Box plots (box, 1st-3rd quartile; horizontal line, median; whiskers, 1.5xIQR); Welch two-sample t-test (two-sided); NS, non-significant (exact p-values provided in **Suppl. Data 1**). Colour codes: eGFP females, pale pink; Egr1 females, bright pink; eGFP males, pale blue; Egr1 males, bright blue. Source data are provided as a Source Data file**.**

Supplementary Figure 5

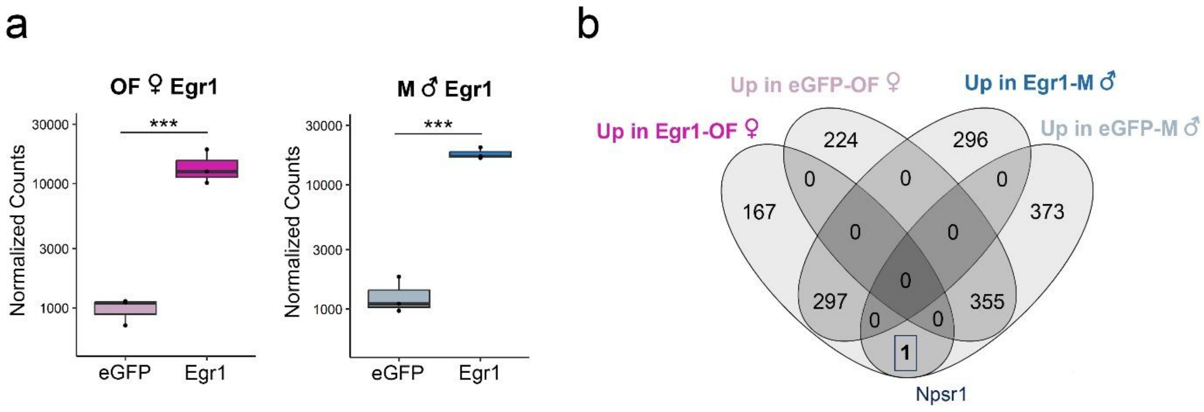

Egr1 overexpression and resulting sex-dependent effects on gene expression in ventral hippocampal neurons. a. Count plots depicting transcript levels of *Egr1* in ventral hippocampal neurons of ovariectomized females (OF, left) and males (M, right) following viral overexpression. **b.** A Venn diagram showing the overlap of differentially expressed genes between Egr1 and eGFP groups in females and males obtained by neuronal (NeuN+) nuclei specific RNA-seq analysis. The box denotes the *Npsr1* gene, which is downregulated in males and upregulated in females after Egr1 overexpression (**Fig. 2f**). RNA-seq data for individual genes is shown using normalized count plots (n=3 biological replicates/group/sex). RNA-seq data were analysed with the Wald statistical test in DESeq2 (two-sided). Box plots (box, 1st-3rd quartile; horizontal line, median; whiskers, 1.5xIQR). ***, $P_{adj}<0.001$; Benjamini-Hochberg correction for multiple testing (exact p-values provided in **Suppl. Data 2**). Colour codes: eGFP females, pale pink; Egr1 females, bright pink; eGFP males, pale blue; Egr1 males, bright blue.

Supplementary Figure 6

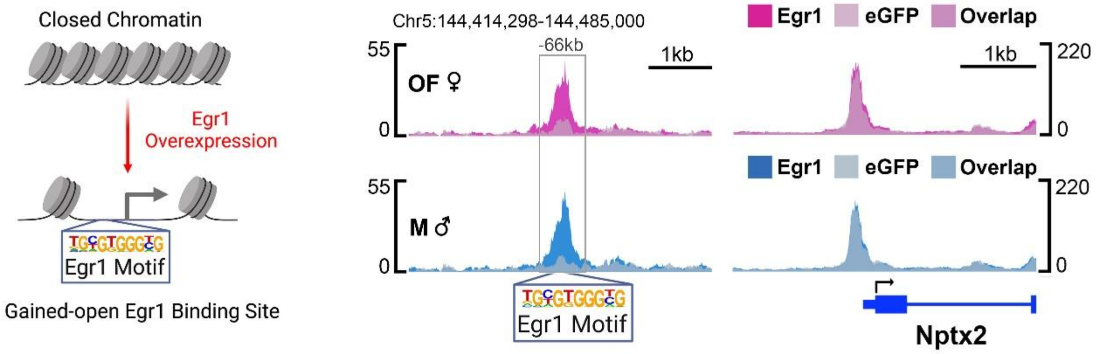

Egr1 gained-open chromatin in ventral hippocampal neurons. A scheme of Egr1-mediated opening of chromatin surrounding the Egr1 motif (left) in ventral hippocampal neurons and an example gene, *Nptx2* (right), which has an Egr1 gained-open putative enhancer region harbouring an Egr1 binding motif in both ovariectomized females (OF, top) and males (M, bottom) 66kb upstream of the TSS. ATAC-seq data is shown using SparK plots of group-average normalized ATAC-seq reads (n = 4 biological replicates/group/sex). ATAC-seq data were analysed using the Wald statistical test in DESeq2 (two-sided). Colour codes: eGFP females, pale pink; Egr1 females, bright pink; eGFP males, pale blue; Egr1 males, bright blue. Schematic (left) was created in BioRender. Rocks, D. (2025) https://BioRender.com/oo9h37f.

Supplementary Figure 7

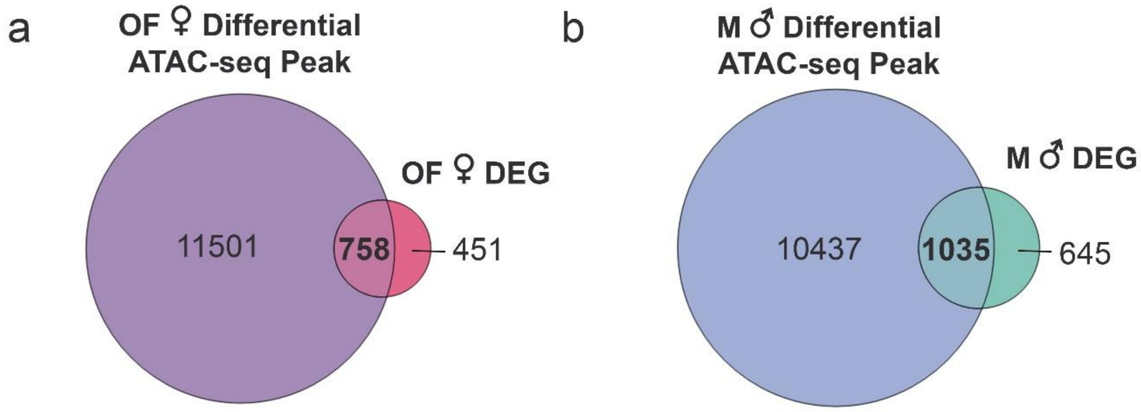

Egr1 has overlapping effects on chromatin accessibility and gene expression in ventral hippocampal neurons. a-b. A Venn diagram showing the overlap of genes annotated to Egr1-induced differential ATAC-seq peaks with Egr1-induced differentially expressed genes (DEGs) in ventral hippocampal neurons of ovariectomized females (OF, **a**) and males (M, **b**).

Supplementary Figure 8

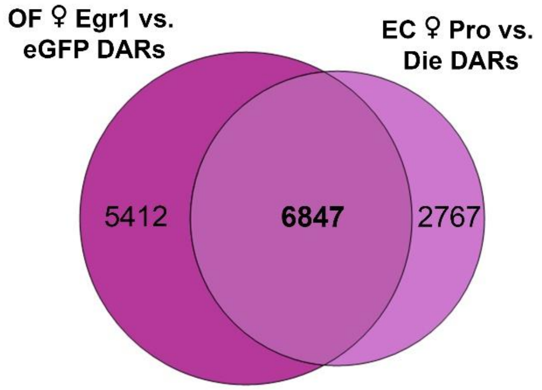

Egr1 induces overlapping chromatin organizational changes in ventral hippocampal neurons with proestrus females. A Venn diagram showing the overlap between genes annotated to female differential ATAC-seq peaks in the Egr1 overexpression and oestrous cycle experiments. OF, ovariectomized female; EC, oestrus cycle; Pro, proestrus; Die, dioestrus.

Supplementary Figure 9

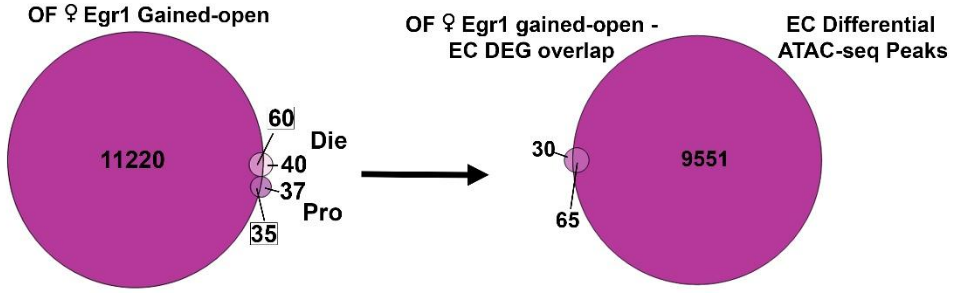

Overlapping elements of neuronal gene regulation by Egr1 after overexpression and across the oestrous cycle. The Venn diagram from **Fig. 4d** showing the overlap between genes annotated to ovariectomized female (OF) Egr1 gained-open regions and differentially expressed genes (DEGs) in ventral hippocampal neurons over the oestrous cycle (left). Taking the overlapping genes from this Venn diagram, an additional Venn diagram depicts that a subset of these genes also undergo chromatin accessibility changes across the oestrous cycle (EC). Pro, proestrus; Die, dioestrus.

Supplementary Figure 10

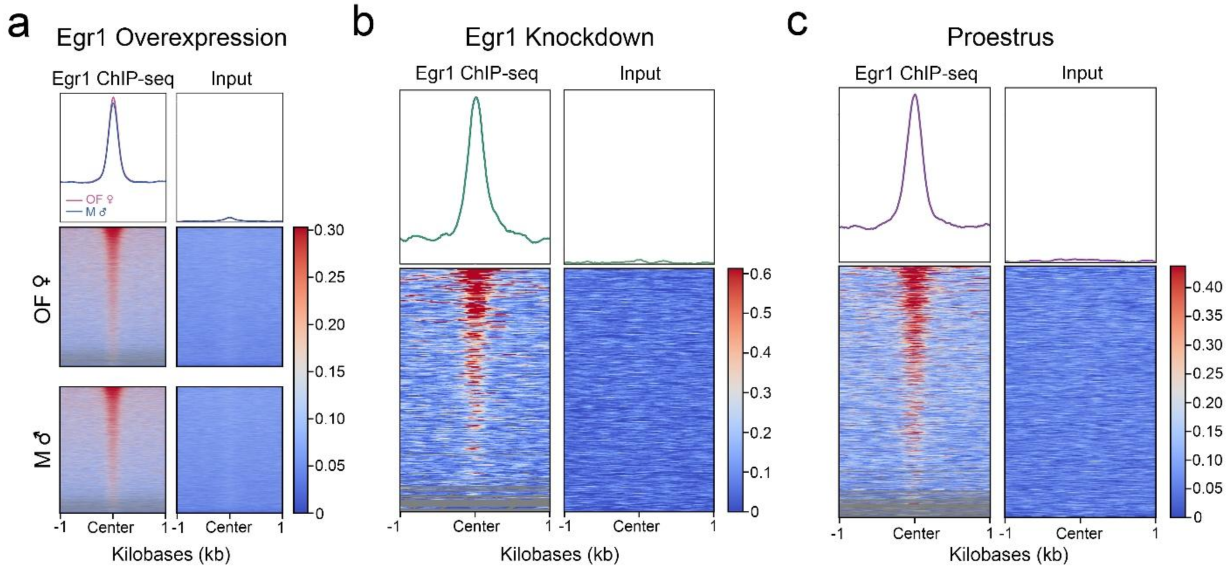

Regions targeted by Egr1 for chromatin regulation are enriched for Egr1 binding activity. Regions of chromatin that gain accessibility after Egr1 overexpression (**a**), lose accessibility after Egr1 knockdown (**b**), or gain accessibility during proestrus compared to dioestrus[18] (**c**), and contain Egr1 binding sites are enriched for Egr1 ChIP-seq signal relative to input control. Egr1 ChIP-seq signal enrichment was determined using previously published data from the male cortex[40].

Supplementary Figure 11

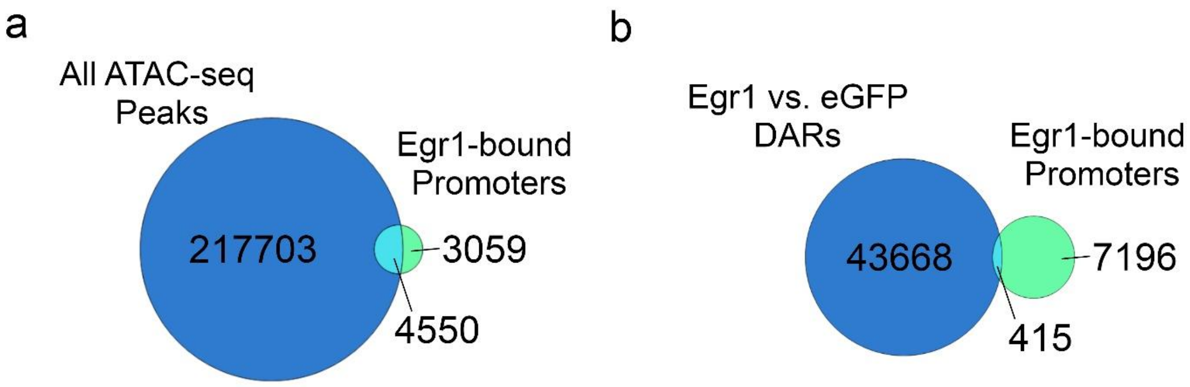

Egr1 enhancers are preferentially enriched in Egr1 vs. eGFP group comparison in ventral hippocampal neurons. The overlap between all unique ATAC-seq peaks (**a**) or Egr1 vs. eGFP differentially accessible regions (DARs; **b**) and Egr1-bound promoters identified in the male cortex by Sun et al.[40]

Supplementary Figure 12

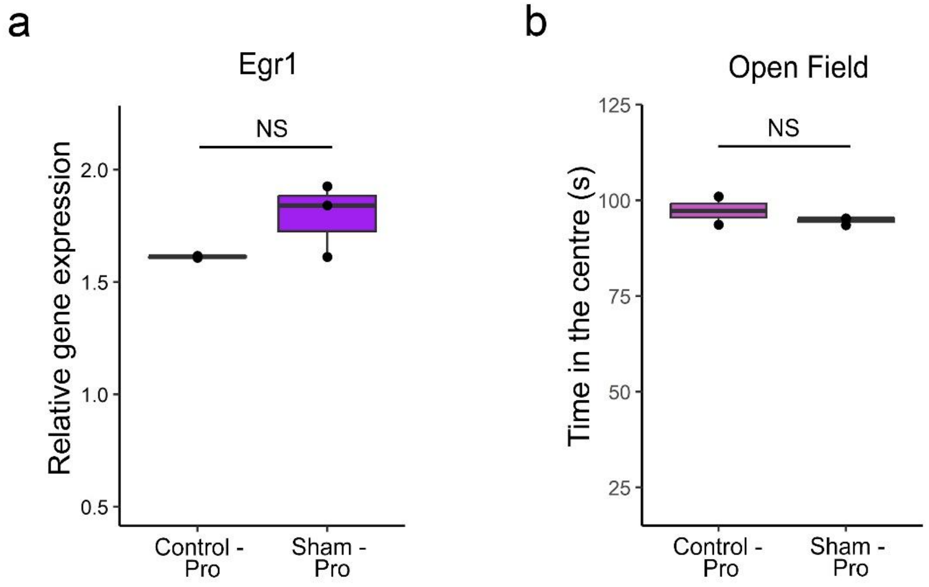

No effect of sham surgery on ventral hippocampal *Egr1* expression and open field behavior. Intact proestrus females that either did (Sham-Pro) or did not (Control-Pro) undergo sham surgery exhibit similar levels of ventral hippocampal *Egr1* expression (**a**) and spend a similar amount of time in the centre time in the open field (**b**, n=2 Control Pro, 3 Sham Pro); analysed with a Welch two-sample t-test (two-sided). NS; non-significant (exact p-values are provided in **Suppl. Data 1**). Source data are provided as a Source Data file**.**

Supplementary Figure 13

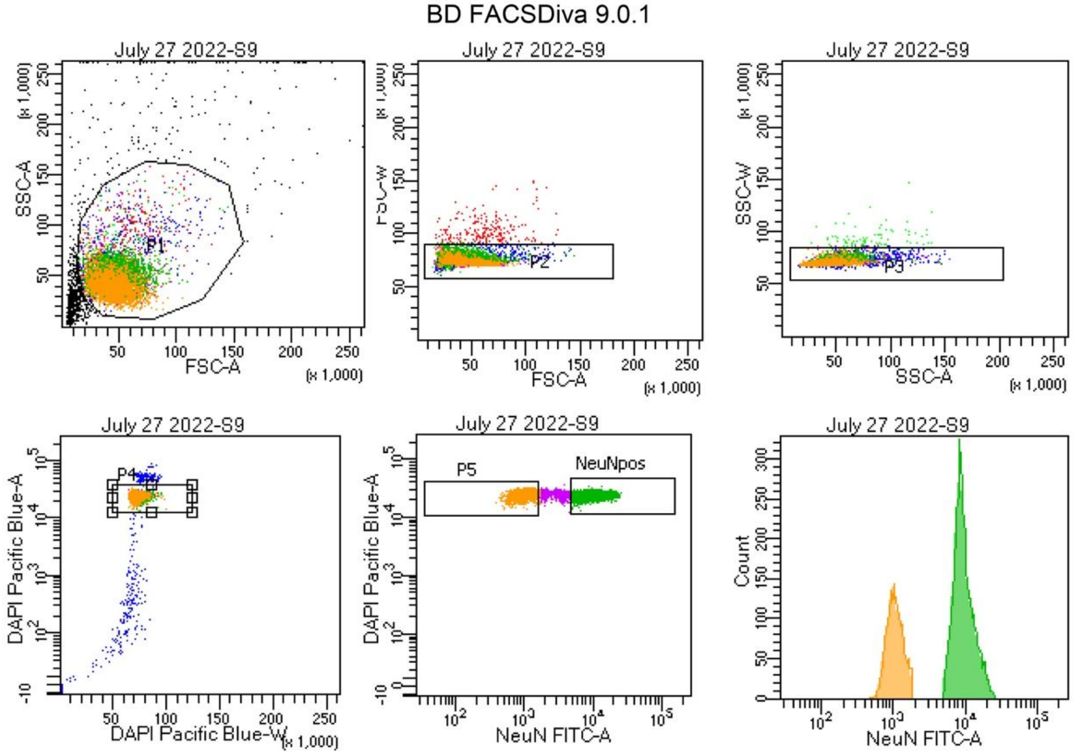

Tube: S9

Population	#Events	%Parent	%Total
All Events	10,000	####	100.0
P1	9,015	90.1	90.1
P2	8,798	97.6	88.0
P3	8,717	99.1	87.2
P4	8,363	95.9	83.6
P5	2,502	29.9	25.0
NeuNpos	5,118	61.2	51.2

Isolation of neuronal nuclei using fluorescence-activated nuclei sorting (FANS). A representative FANS report which demonstrates the gating strategy used to: 1) separate the nuclei populations from debris (gates P1-P3); 2) sort only single nuclei, based on the DAPI fluorescent signal (gate P4); and, lastly, 3) separate NeuN+ (neuronal) nuclei (gate P6) from NeuN- (non-neuronal) nuclei (gate P5).

Supplementary Figure 14

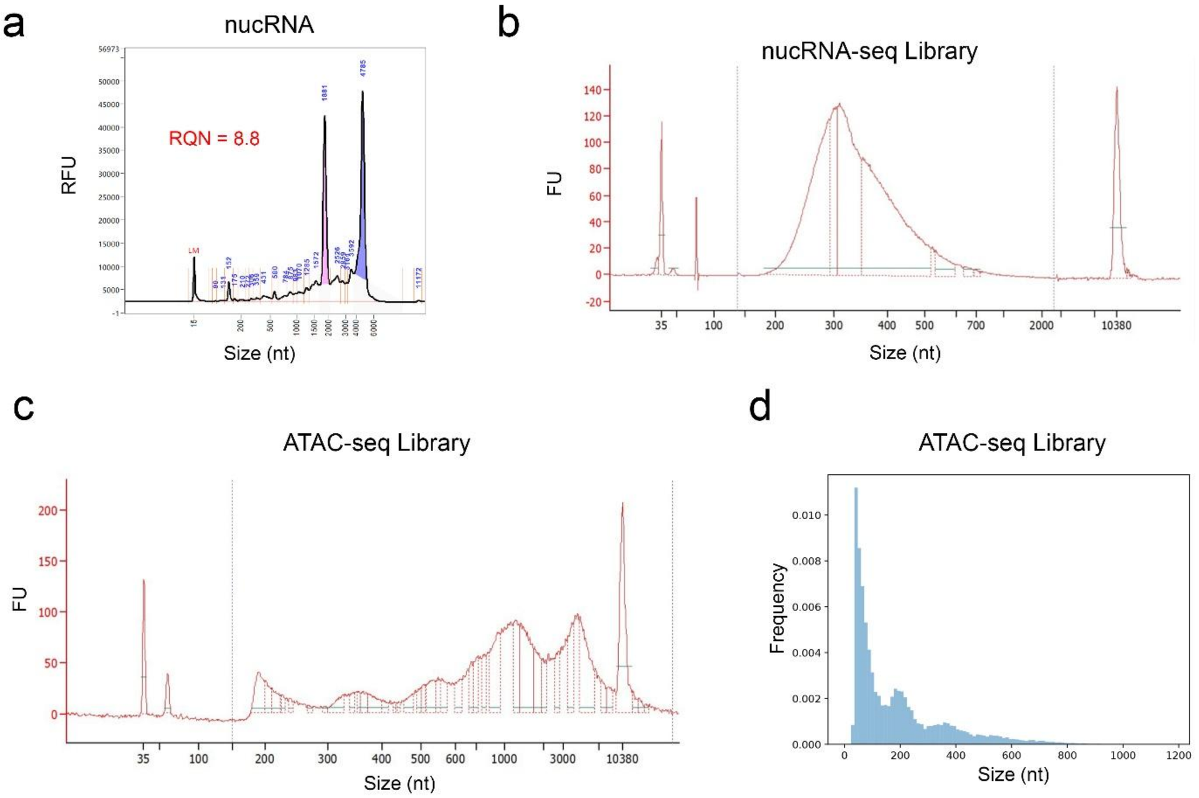

Quality control for nucRNA and sequencing libraries. a. A characteristic Fragment Analyzer trace of a nuclear RNA (nucRNA) sample used in this study, with an RQN > 7 demonstrating high-quality RNA. Bioanalyzer traces of a cDNA library prepared from nucRNA (**b**) and from an ATAC-seq library (**c**) used in this study, with the ATAC-seq trace exhibiting a characteristic pattern of nucleosomal periodicity. **d.** A fragment distribution plot for the same ATAC-seq library shown in (**c**), demonstrating that insert sizes correspond primarily to nucleosome-free, mono-, and di-nucleosomal DNA. RFU, relative fluorescence units; FU, fluorescence units; nt, nucleotides.
