# Supplementary material for: Egr1 is a sex-dependent regulator of neuronal chromatin, structural plasticity, and behaviour
Source: Nat Commun. 2025 Dec 13;16:11405. doi: 10.1038/s41467-025-66217-6 (PMC12738549; doi:10.1038/s41467-025-66217-6)
Supplement: Supplementary file 2 — Description of Additional Supplementary Files [file 41467_2025_66217_MOESM2_ESM.pdf]

Descriptions of Additional Supplementary Data Files

Supplementary Data 1: Summary statistics for ANOVA and T-tests

Supplementary Data 2: Differentially expressed genes from the Egr1 overexpression RNA-seq analysis

Supplementary Data 3: Results from the Egr1 overexpression GSEA enrichment analysis

Supplementary Data 4: Differentially accessible regions from the ATAC-seq Egr1 overexpression experiment

Supplementary Data 5: Results for the Egr1 overexpression Homer motif analysis

Supplementary Data 6: Differentially expressed genes from the Egr1 knockdown RNA-seq analysis and enriched estrogen-response genes

Supplementary Data 7: Results from the Egr1 knockdown GSEA enrichment analysis

Supplementary Data 8: Differentially accessible regions from the Egr1 knockdown ATAC-seq experiment and enriched estrogen-response genes

Supplementary Data 9: Results for the Egr1 knockdown Homer motif analysis

Supplementary Data 10: RNA-seq data basic information for the overexpression and knockdown experiments

Supplementary Data 11: ATAC-seq data basic information for the overexpression and knockdown experiments
