# Supplementary material for: Egr1 is a sex-dependent regulator of neuronal chromatin, structural plasticity, and behaviour
Source: Nat Commun. 2025 Dec 13;16:11405. doi: 10.1038/s41467-025-66217-6 (PMC12738549; doi:10.1038/s41467-025-66217-6)
Supplement: Supplementary file 14 — Reporting Summary [file 41467_2025_66217_MOESM14_ESM.pdf]

nature portfolio

Corresponding author(s): Marija Kundakovic

Last updated by author(s): Sep 24, 2025

Reporting Summary

Nature Portfolio wishes to improve the reproducibility of the work that we publish. This form provides structure for consistency and transparency in reporting. For further information on Nature Portfolio policies, see our Editorial Policies and the Editorial Policy Checklist.

Statistics

For all statistical analyses, confirm that the following items are present in the figure legend, table legend, main text, or Methods section.

n/a	Confirmed	
☐	☒	The exact sample size (*n*) for each experimental group/condition, given as a discrete number and unit of measurement
☐	☒	A statement on whether measurements were taken from distinct samples or whether the same sample was measured repeatedly
☐	☒	The statistical test(s) used AND whether they are one- or two-sided<br>*Only common tests should be described solely by name; describe more complex techniques in the Methods section.*
☐	☒	A description of all covariates tested
☐	☒	A description of any assumptions or corrections, such as tests of normality and adjustment for multiple comparisons
☐	☒	A full description of the statistical parameters including central tendency (e.g. means) or other basic estimates (e.g. regression coefficient) AND variation (e.g. standard deviation) or associated estimates of uncertainty (e.g. confidence intervals)
☐	☒	For null hypothesis testing, the test statistic (e.g. *F*, *t*, *r*) with confidence intervals, effect sizes, degrees of freedom and *P* value noted<br>*Give P values as exact values whenever suitable.*
☒	☐	For Bayesian analysis, information on the choice of priors and Markov chain Monte Carlo settings
☒	☐	For hierarchical and complex designs, identification of the appropriate level for tests and full reporting of outcomes
☐	☒	Estimates of effect sizes (e.g. Cohen's *d*, Pearson's *r*), indicating how they were calculated

Our web collection on statistics for biologists contains articles on many of the points above.

Software and code

Policy information about availability of computer code

Data collection	Behavioural data were collected using the ANY-maze Video Tracking Software (ANY-maze 5.1, Stoelting Co). Real-time qRT-PCR data were collected using QuantStudio™ Design and Analysis Desktop Software version 1.4 (Applied Biosystems; Thermo Fisher Scientific, Waltham, MA). Microscopy data were colected using NIS-Elements Basic Research (Nikon). Flow cytometry data were collected using BD FACSDiva v8.0.1 software.
Data analysis	Flow cytometry data were analyzed using BD FACSDiva v8.0.1 software.<br>We used the following packages and software for bioinformatic analysis:<br>R (version 4.2.2, https://cran.r-project.org/); BWA (version 0.7.12, http://bio-bwa.sourceforge.net/); DESeq2 (version 1.36, https://bioconductor.org/packages/release/bioc/html/DESeq2.html); EnrichR (version 3.2, https://cran.r-project.org/web/packages/enrichR/vignettes/enrichR.html); SparK (version 2.6.2, https://github.com/harbourlab/SparK); STAR (version 2.7.10, https://github.com/alexdobin/STAR); EnhancedVolcano (version 1.14, https://github.com/kevinblighe/EnhancedVolcano); GSEA (version 4.3.2, https://www.gsea-msigdb.org/gsea/index.jsp); Cytoscape (version 3.10.1, https://cytoscape.org/); MACS2 (version 2.2, https://pypi.org/project/MACS2/); ChIPpeakAnno (version 3.37.2, https://github.com/jianhong/ChIPpeakAnno); Homer (version 4.11, http://homer.ucsd.edu/homer/index.html); DiffBind (version 3.20, https://bioconductor.org/packages/release/bioc/html/DiffBind.html).<br>Dendritic spine density measurements were determined using ImageJ v1.53 (public domain software from the National Institutes of Health; http://imagej.nih.gov/ij/).

For manuscripts utilizing custom algorithms or software that are central to the research but not yet described in published literature, software must be made available to editors and reviewers. We strongly encourage code deposition in a community repository (e.g. GitHub). See the Nature Portfolio guidelines for submitting code & software for further information.

Data

Policy information about availability of data

All manuscripts must include a data availability statement. This statement should provide the following information, where applicable:

- Accession codes, unique identifiers, or web links for publicly available datasets
- A description of any restrictions on data availability
- For clinical datasets or third party data, please ensure that the statement adheres to our policy

RNA-seq and ATAC-seq data are available from the NCBI Gene Expression Omnibus database under accession GSE249978.
ChIP-seq data for H3K4me1, H3K27ac, and H3K4me3 in neuronal CA1 neurons of male mice was obtained from GSE74964.
ChIP-seq data for Egr1 in the male prefrontal cortex was obtained from GSE108768.
RNA-seq and ATAC-seq data from vHIP neurons of proestrus females, dioestrus females, and males which we previously published (Jaric et al. Nature Communications 2019) are available at GSE114036.
Source data are provided with this paper. Any other relevant data supporting the key findings of this study are available within the article or from the corresponding author upon request.

Research involving human participants, their data, or biological material

Policy information about studies with human participants or human data. See also policy information about sex, gender (identity/presentation), and sexual orientation and race, ethnicity and racism.

Reporting on sex and gender	*Use the terms sex (biological attribute) and gender (shaped by social and cultural circumstances) carefully in order to avoid confusing both terms. Indicate if findings apply to only one sex or gender; describe whether sex and gender were considered in study design; whether sex and/or gender was determined based on self-reporting or assigned and methods used.*<br>*Provide in the source data disaggregated sex and gender data, where this information has been collected, and if consent has been obtained for sharing of individual-level data; provide overall numbers in this Reporting Summary. Please state if this information has not been collected.*<br>*Report sex- and gender-based analyses where performed, justify reasons for lack of sex- and gender-based analysis.*
Reporting on race, ethnicity, or other socially relevant groupings	*Please specify the socially constructed or socially relevant categorization variable(s) used in your manuscript and explain why they were used. Please note that such variables should not be used as proxies for other socially constructed/relevant variables (for example, race or ethnicity should not be used as a proxy for socioeconomic status).*<br>*Provide clear definitions of the relevant terms used, how they were provided (by the participants/respondents, the researchers, or third parties), and the method(s) used to classify people into the different categories (e.g. self-report, census or administrative data, social media data, etc.)*<br>*Please provide details about how you controlled for confounding variables in your analyses.*
Population characteristics	*Describe the covariate-relevant population characteristics of the human research participants (e.g. age, genotypic information, past and current diagnosis and treatment categories). If you filled out the behavioural & social sciences study design questions and have nothing to add here, write "See above."*
Recruitment	*Describe how participants were recruited. Outline any potential self-selection bias or other biases that may be present and how these are likely to impact results.*
Ethics oversight	*Identify the organization(s) that approved the study protocol.*

Note that full information on the approval of the study protocol must also be provided in the manuscript.

Field-specific reporting

Please select the one below that is the best fit for your research. If you are not sure, read the appropriate sections before making your selection.

☒ Life sciences ☐ Behavioural & social sciences ☐ Ecological, evolutionary & environmental sciences

For a reference copy of the document with all sections, see nature.com/documents/nr-reporting-summary-flat.pdf

Life sciences study design

All studies must disclose on these points even when the disclosure is negative.

Sample size	No statistical methods were used to determine sample size. Sample sizes were chosen based on previous experience and published work (PMID: 35705546, PMID:31253786, PMID:22955991). The overexpression RNA-seq experiment included 3 biological replicates (pooled from two animals) per group per sex. The overexpression ATAC-seq experiment included 4 biological replicates per group per sex. The knockdown RNA-seq and ATAC-seq experiments included 3 biological replicates per group per sex. Behavioural experiments included 5-12 animals per group. Dendritic spine density was measured from 5 animals per group per sex, with 4 images taken from each animal and 4 dendrite segments sampled from each images, yielding 80 spine density measurement per group per sex.
Data exclusions	Animals were excluded from the analysis of behavioural data in the Egr1 overexpression experiment if either no viral expression was present or if there was evidence of off-target expression or tissue damage (this occurred in n=4 females and n=2 males, or 12.5% of animals tested).

	Intact cycling females used as a proestrus comparison for the cyclical oestradiol treatment experiment were excluded if they were not in the proestrus stage during behavioral testing or tissue collection, as determined by vaginal smear cytology.
Replication	Since these were animal studies, we used biological replicates in all experiments. For the overexpression RNA-seq experiment and the knockdown ATAC-seq and RNA-seq experiments we used 3 biological replicates per group per sex. For the ATAC-seq experiment we used 4 biological replicates per group per sex. For the Golgi-Cox staining experiment we used 5 biological replicates per group per sex. For behavioural analyses, we used 8-12 animals per group.
Randomization	For overexpression and knockdown experiments, cages (n=3-5/cage) were randomly selected to receive either the experimental or control virus. No randomization was performed for oestrus cycle experiments, since these were dependent on oestrus cycle predictions and the cycle length of individual animals.
Blinding	Imaging and analysis of dendritic spine density was performed while blinded to the experimental condition.

Reporting for specific materials, systems and methods

We require information from authors about some types of materials, experimental systems and methods used in many studies. Here, indicate whether each material, system or method listed is relevant to your study. If you are not sure if a list item applies to your research, read the appropriate section before selecting a response.

Materials & experimental systems

n/a	Involved in the study
☐	☒ Antibodies
☒	☐ Eukaryotic cell lines
☒	☐ Palaeontology and archaeology
☐	☒ Animals and other organisms
☒	☐ Clinical data
☒	☐ Dual use research of concern
☒	☐ Plants

Methods

n/a	Involved in the study
☒	☐ ChIP-seq
☐	☒ Flow cytometry
☒	☐ MRI-based neuroimaging

Antibodies

Antibodies used	Antibodies for FACS: Mouse monoclonal Anti-NeuN Antibody, clone A60, conjugated to AlexaFluor 488 (MAB377X; Millipore, 1:1000); and Mouse monoclonal IgG1-k, clone MOPC-21 antibody control, conjugated to Alexa Fluor 488 (FCMAB310A4, Millipore, 1:1000).<br>Primary antibody for immunofluorescence analysis: rabbit monoclonal anti-Egr1 antibody (Cell Signaling, 4154, 1:500).<br>Secondary antibody for immunofluorescence analysis: donkey anti-rabbit IgG conjugated to AlexaFluor-594 (Invitrogen, A-21207, 1:250).
Validation	The anti-NeuN Alexa-488 FACS antibody (MAB377X; Millipore) has been validated for fluorescence-activated nuclei sorting (PMID:27113501, PMID:31253786).<br>We previously validated the Isotype control (FCMAB310A4, Millipore) as an appropriate negative control for our FACS sorting (PMID:31253786, PMID: 35705546).<br>Rabbit monoclonal anti-Egr1 antibody (Sigma-Aldrich, 06-935) was validated by Western Blot and confocal microscopy of PC12 cells treated with NGF by the manufacturer.<br>Donkey anti-rabbit IgG conjugated to AlexaFluor-594 (Invitrogen, A-21207) was verified for immunostaining by the manufacturer and numerous publications (e.g. PMID: 27197019)

Animals and other research organisms

Policy information about studies involving animals; ARRIVE guidelines recommended for reporting animal research, and Sex and Gender in Research

Laboratory animals	All animals used in this study were C57BL6/J mice and arrived from Jackson Laboratories at 5-6 weeks of age. Ovariectomized females in cohort 2 and cohorts 4-6 underwent ovariectomy at 4 weeks of age at Jackson Laboratories and recovered for 1 week prior to arriving, while ovariectomized females in cohort 3 underwent ovariectomy in-house at 8 weeks of age (the relationship between animal cohort and the data presented are described in detail in the Methods section under the Animals heading). All animals were housed in same-sex cages (n=3-5) and were allowed to habituate for 2 weeks to the facility prior to experiments. All behavioural and molecular experiments were performed on mice 10-15 weeks of age. Mice were kept on a 12:12h light:dark cycle (lights on at 8 a.m.) and were given ad libitum access to food and water. The temperature of the room is maintained at 21°C and the humidity ranges from 30-70%.
Wild animals	The study did not involve wild animals.
Reporting on sex	Examining sex differences was a major focus of this study and therefore both males and females were included in all of the overexpression and knockdown experiments. Notably, for overexpression experiments, females were ovariectomized to remove the confounding effects of naturally cycling Egr1 levels, and therefore may not be suitable for direct comparison to males who were gonadally intact. For this reason we did not directly assess sex differences in the overexpression experiments. Rather, the effect of

Egr1 was determined within each sex independently and indirectly compared. For knockdown experiments, which included intact males and females, sex was included as a variable in the analysis and group * sex interactions were evaluated in both the RNA-seq and ATAC-seq data.

Field-collected samples	The study did not involve field-collected samples.
Ethics oversight	All animal procedures were approved by the Institutional Animal Care and Use Committee at Fordham University.

Note that full information on the approval of the study protocol must also be provided in the manuscript.

Plants

Seed stocks	*Report on the source of all seed stocks or other plant material used. If applicable, state the seed stock centre and catalogue number. If plant specimens were collected from the field, describe the collection location, date and sampling procedures.*
Novel plant genotypes	*Describe the methods by which all novel plant genotypes were produced. This includes those generated by transgenic approaches, gene editing, chemical/radiation-based mutagenesis and hybridization. For transgenic lines, describe the transformation method, the number of independent lines analyzed and the generation upon which experiments were performed. For gene-edited lines, describe the editor used, the endogenous sequence targeted for editing, the targeting guide RNA sequence (if applicable) and how the editor was applied.*
Authentication	*Describe any authentication procedures for each seed stock used or novel genotype generated. Describe any experiments used to assess the effect of a mutation and, where applicable, how potential secondary effects (e.g. second site T-DNA insertions, mosiacism, off-target gene editing) were examined.*

Flow Cytometry

Plots

Confirm that:

- [x] The axis labels state the marker and fluorochrome used (e.g. CD4-FITC).
- [x] The axis scales are clearly visible. Include numbers along axes only for bottom left plot of group (a 'group' is an analysis of identical markers).
- [x] All plots are contour plots with outliers or pseudocolor plots.
- [x] A numerical value for number of cells or percentage (with statistics) is provided.

Methodology

Sample preparation	Mice were sacrificed and bilateral ventral hippocampi were rapidly dissected on ice then frozen in liquid nitrogen. Nuclei were extracted from frozen brain tissue using ultracentrifugation through a sucrose gradient. Neuronal (NeuN+) nuclei were purified by fluorescence-activated nuclei sorting using an anti-NeuN antibody conjugated to AlexaFluor-488 on a BD FACSAria instrument (Suppl. Fig. 13).
Instrument	FACS Aria instrument (BD)
Software	BD FACSDiva v8.0.1 software
Cell population abundance	For overexpression RNA-seq, we collected 142,041-250,000 NeuN+ nuclei per biological replicate. For overexpression ATAC-seq, we collected 50,000 NeuN+ nuclei per biological replicate. For the knockdown experiments, we first collected 50,000 NeuN+ nuclei per replicate for ATAC-seq and then collected the remaining nuclei for RNA-seq.
Gating strategy	We set the gates using 3 controls: NeuN+ only, NeuN-isotype with DAPI, DAPI-only. The gating strategy allowed us to separate the nuclei populations from debris, purify single nuclei (based on the DAPI), and to separate NeuN+ (neuronal) nuclei from NeuN- (non-neuronal) nuclei (based on Alexa-488 signal).

- [x] Tick this box to confirm that a figure exemplifying the gating strategy is provided in the Supplementary Information.
